# Supplementary material for: Genotype–Phenotype Correlation in a Large Cohort of Eastern Sicilian Patients Affected by Phenylketonuria: Newborn Screening Program, Clinical Features, and Follow-Up
Source: Nutrients. 2025 Jan 21;17(3):379. doi: 10.3390/nu17030379 (PMC11819930; doi:10.3390/nu17030379)
Supplement: Supplementary file 1 [file nutrients-17-00379-s001.zip › nutrients-3402334-supplementary.pdf]

Table S1. T-test comparison of Phe/Tyr ratios between different phenotypic classes. Data are presented for the pairwise comparisons between Classic, Mild, and Moderate phenotypes. None of the comparisons showed statistically significant differences ($p > 0.05$), indicating similar Phe/Tyr ratios across the phenotypic classes. *Legend*: **SE**: Standard Error; **df**: Degrees of Freedom.

**Phenotype**		**Phenotype**	**Mean Difference**	**SE**	**df**	**t-value**	***p*-value**
Classic	vs.	Mild	0.0157	0.390	277	0.040	0.999
Classic	vs.	Moderate	0.3407	0.413	277	0.824	0.688
Mild	vs	Moderate	0.3251	0.393	277	0.827	0.686

Table S2. T-test and group descriptives on adherence to diet in classic PKU patients N=number of Phe/tyr ratio measurements. SD=standard deviation, SE=standard error.

Independet Samples T-Test								
		Statistic	df	*p*	Mean difference	SE difference		Effect Size
Phe/Tyr	Student's t	−12.9	121	< 0.001	−12.6	0.975	Cohen's d	−2.63
Group Descriptives								
		Group	N		Mean	Median	SD	SE
Phe/Tyr		compliant	90		4.77	3.69	3.77	0.397
		not compliant	33		17.4	18.3	6.88	1.2

Table S3. T-test and group descriptives on adherence to diet in classic PKU patients under and above six years of age. N=number of Phe/tyr ratio measurements. SD=standard deviation SE=standard error.

Independent Samples T-Test								
		Statistic	df	*p*	Mean difference	SE difference		Effect Size
Phe/Tyr	Student's t	−2.64	88.0	0.010	−2.04	0.774	Cohen's d	−0.560
Group Descriptives								
	Group		N		Mean	Median	SD	SE
Phe/Tyr	pre-scholar		50		3.86	2.65	3.64	0.515
	scholar/adult		40		5.9	5.33	3.65	0.578
